# Impact of systemic therapy on circulating leukocyte populations in patients with metastatic breast cancer

**DOI:** 10.1038/s41598-019-49943-y

**Published:** 2019-09-17

**Authors:** Anna-Maria Larsson, Anna Roxå, Karin Leandersson, Caroline Bergenfelz

**Affiliations:** 1 Department of Clinical Sciences Lund, Division of Oncology and Pathology, Lund University, Lund, Sweden; 2 Department of Hematology, Oncology and Radiation Physics, Skåne University Hospital, Lund, Sweden; 3 Department of Translational Medicine, Cancer Immunology, Lund University, Malmö, 21428 Sweden; 4 Department of Translational Medicine, Experimental Infection Medicine, Lund University, Malmö, 21428 Sweden

**Keywords:** Breast cancer, Tumour immunology

## Abstract

Tumors affect the immune system, locally and systemically. The frequencies of specific circulating immune cell populations correlate with disease progression as well as prognosis of the patients. Although largely neglected, conventional antitumoral therapies often possess immunomodulatory properties and affect the levels of specific immune cell populations. Most information, however, derive from animal or *in vitro* studies. As this could impact prognosis as well as response to therapy, further studies of the effects of treatment on circulating immune cells in patients are warranted. In this pilot study, we evaluated a wide panel of circulating immune cells over time (up to six months) in ten patients with metastatic breast cancer receiving standard antitumoral regimens. Overall, endocrine therapy tends to enrich for natural killer (NK) and natural killer T (NKT) cells in the circulation, whereas both chemotherapy and endocrine therapy reduce the levels of circulating monocytic myeloid-derived suppressor cells (Mo-MDSCs). This indicates that the systemic immunosuppressive profile observed in patients tends to revert over the course of systemic therapy and holds promise for future combination treatment with standard antitumoral agents and immunotherapy.

## Introduction

Breast cancer is the most common cancer in women worldwide, with more than 2.4 million new cases annually^1^. Even though advances in early detection and treatment strategies have led to improved survival, specifically in high-income countries, breast cancer remains the most common cause of cancer-related death in women globally^1–3^.

Even though adjuvant treatment regimens have improved, about 20–30% of women with initially regional disease will develop metastatic disease^4,5^. For metastatic disease, there is in most cases no cure and the main goals of treatment are improving quality of life, symptom prevention and palliation, and survival prolongation^6^. The medical treatment options include chemotherapy, endocrine therapy, targeted therapies and supportive care measures. International consensus guidelines for treatment of metastatic breast cancer (MBC) were finally published in 2012 and are updated biannually^7^, but treatment choices are still complex and based on factors related to the disease and to the patient. Most patients with metastatic disease will receive endocrine therapy (ER positive tumors) or chemotherapy (triple negative breast cancer; TNBC and aggressive disease), whereas HER2-directed therapy in combination with chemotherapy or endocrine therapy is the basis for HER2-positive disease^7^. In recent years, new targeted therapies have also been introduced for subgroups of MBC patients, such as cycline-dependent-kinase inhibitors (CDK-I) in combination with endocrine therapy in ER positive disease^8,9^ and currently, check-point inhibitors are being introduced in clinical practice for TNBC^10^. However, at the time of the current study, no patient received any additional systemic targeted therapy besides HER2-directed therapy, chemotherapy or endocrine therapy.

During the past decades, the importance of various immune cell populations in tumor development, progression and treatment resistance has been recognized^11^. On one hand, immune cells such as cytotoxic T cells (CTLs), natural killer (NK) cells, NKT cells and pro-inflammatory neutrophils or macrophages (N1 neutrophils and M1 macrophages) exert direct cytotoxic effect on tumor cells^12^. On the other hand, regulatory T cells (Tregs), Th2-skewed T helper cells (Th), anti-inflammatory neutrophils or macrophages (N2 neutrophils and M2 macrophages) and myeloid-derived suppressor cells (MDSCs) are involved in immunosuppression, tissue remodeling and angiogenesis, and are thus regarded pro-tumorigenic^12,13^. Today it is a well-documented fact that tumors actively modulate the immune system towards a more tumor-permissive phenotype, locally as well as systemically. Leukocytosis, lymphopenia and neutrophilia are common hematological findings that correlate with disease progression, and consequently poor prognosis, in several malignancies^14–16^. In breast cancer patients, similar systemic immune alterations are detected. Decreased levels of circulating lymphocytes (especially CD8^+^ CTLs) and myeloid dendritic cells (mDCs) or increased levels of monocytes, NK cells, Tregs and MDSCs correlate with stage and/or disease progression^17–23^.

Although conventional antitumoral treatments target the malignant cells, some approaches also have immunomodulatory properties^24–26^. Substantial efforts are made to investigate the efficacy of immunotherapy in breast cancer and recently a clinical trial showed prolonged progression-free survival in patients with advanced TNBC treated with the PD-L1 antibody atezolizumab in combination with nab-paclitaxel^10^. As the levels of various immune cell populations could impact prognosis and response to therapy, and the fact that immunotherapies most often is given in combination with conventional therapy, further studies of the effects of standard treatment on circulating immune cells in patients are warranted.

In this pilot study, we prospectively monitored patients with MBC to evaluate whether specific circulating leukocyte populations, including T lymphocytes, NK cells and monocytic-MDSCs (Mo-MDSCs) in peripheral blood are affected during standard systemic therapies.

## Results

### Patient characteristics

A total of ten female patients with MBC were included in this study (Table 1). Median age was 58 years (range 44–77 years). Three patients had *de novo* metastatic disease, whereas seven patients were diagnosed with distant recurrence. Four patients had more than three metastatic loci and five patients had visceral metastasis. Eight patients had ER +/HER2-tumors, one had ER +/HER2+ disease and one patient had TNBC. Among the eight patients with ER +/HER2- disease, five received endocrine therapy (ET; two patients received tamoxifen and three patients aromatase inhibitors) and three patients received chemotherapy. Chemotherapy regimens used were FEC (5-fluorouracil [5-FU], epirubicin, cyclophosphamide) in two patients and docetaxel in one patient with ER +/HER2- MBC. The patient with ER +/HER2+ disease received trastuzumab in combination with capecitabine and the patient with TNBC was treated with capecitabine. One patient was diagnosed with early progression at first evaluation (after 3 months of endocrine therapy) whereas the mean progression-free survival (PFS) was 23 months (range 2.8–56.7 months). See Table 1 for specification of treatment regimens and clinical information.

**Table 1:** Patient/tumor characteristics and treatment.

Patient	Age	Subtype	MFI (y)	Metastatic sites (no)	PFS (mos)	Treatment	Treatment classification
Pat 1	44	ER +/HER2−	0	≥3	5.5	FEC	ChT
Pat 2	72	ER +/HER2−	6.2	<3	2.8	TAM	ET
Pat 3	71	ER +/HER2−	19.1	<3	33.6	AI	ET
Pat 4	58	ER +/HER2−	0	<3	22.3	AI	ET
Pat 5	45	ER +/HER2−	0	<3	29.9	TAM	ET
Pat 6	63	ER +/HER2−	4.5	≥3	20.9	FEC	ChT
Pat 7	53	ER +/HER2−	3.8	<3	33.9	AI	ET
Pat 8	77	TNBC	1.0	≥3	16.0	CAPE + RT	ChT + RT
Pat 9	56	ER +/HER2+	4.8	<3	>56.7	CAPE + trastuzumab + RT	ChT + RT
Pat 10	50	ER +/HER2−	5.1	≥3	8.5	docetaxel	ChT

Abbreviations: ER, estrogen receptor; HER2, human epidermal growth factor receptor 2; TNBC, triple negative breast cancer; MFI, metastasis free interval (years); PFS, progression-free survival (months); FEC, 5-fluorouracil (5-FU), epirubicin and cyclophosphamide; TAM, tamoxifen; AI, aromatase inhibitor; CAPE, capecitabine; RT, radiotherapy; ChT, chemotherapy; and ET, endocrine therapy.

### Modest alterations in circulating T cell populations in patients receiving systemic therapy

In order to investigate whether systemic treatment affect circulating immune cell populations, we collected venous blood at baseline (BL; *i*.*e*. before onset of treatment) and after one and three months of treatment. Freshly isolated peripheral blood mononuclear cells (PBMCs) were analyzed using flow cytometry and variations in specific immune cell populations as compared to BL assessed. Representative dot plots and gating strategies of all cell populations are depicted in Supplementary Fig. 1.

**Figure 1:**
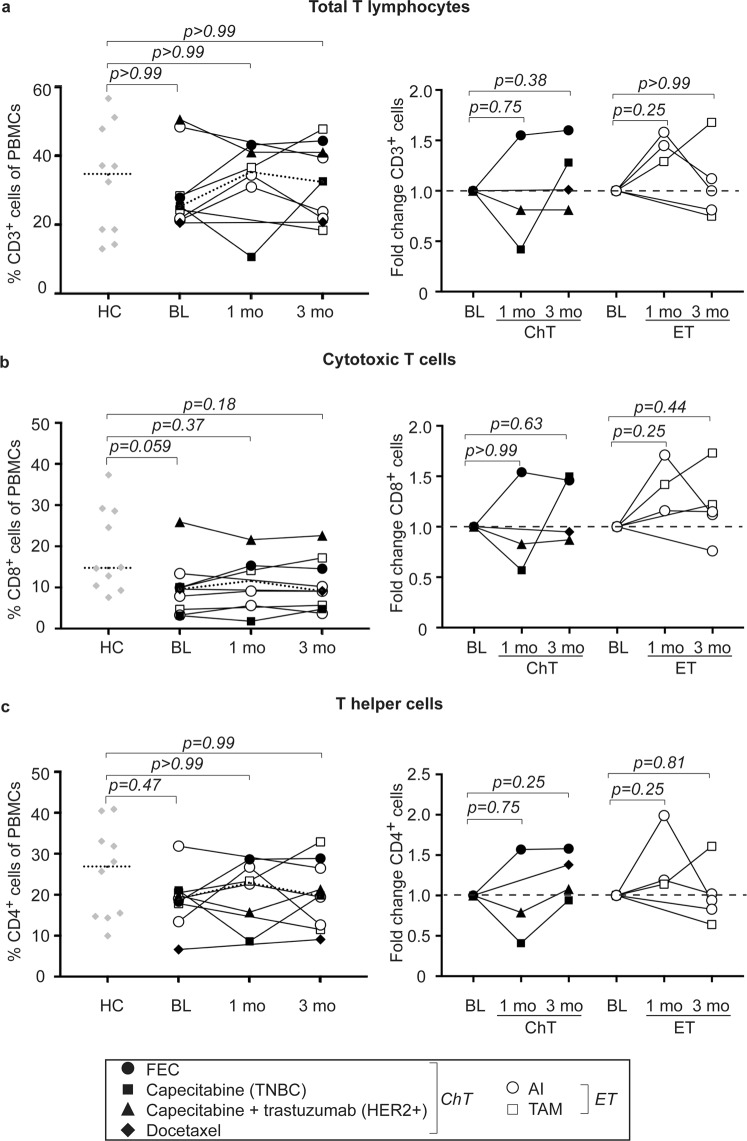
T lymphocytes are modestly affected during systemic treatment. Flow cytometric analyzes of peripheral blood mononuclear cells (PBMCs) from healthy controls (HC) and breast cancer patients before (baseline; BL), at 1 month or at 3 months of treatment (see Supplementary Fig. 1 and Table 1 for specification of treatment and clinical information). (a–c, *left panels*) Percentages of total CD3^+^ T cells (**a**) CD8^+^ T_c_/CTLs (**b**) or CD4^+^ Th cells (**c**) of PBMCs are depicted over time for individual patients. Dashed lines represent median. HC *n* = 10, BL *n* = 9, 1mo *n* = 6 and 3mo *n* = 9. Exact p-values, by Kruskal-Wallis with Dunn’s multiple comparison test, are indicated. (a–c, *right panels*) Fold change as compared to BL of total CD3^+^ T cells (**a**), CD8^+^ T_c_/CTLs (**b**) or CD4^+^ Th (**c**) upon treatment with chemotherapy (ChT, black symbols; BL *n* = 4, 1mo *n* = 3, 3 mo *n* = 4) or endocrine therapy (ET, white symbols; BL *n* = 5, 1mo *n* = 3, 3mo *n* = 5). Dashed line represents BL levels (set to 1). Exact p-values, by Wilcoxon signed-rank test, are indicated.

Compared to healthy donors, patients with MBC generally display slightly reduced levels of T lymphocytes at baseline (Fig. 1, left panels). Overall, endocrine therapy (white symbols in figures) tends to induce a transient increase in both total T lymphocytes (CD3^+^, Fig. 1a), cytotoxic- (CD8^+^ T_c_/CTLs, Fig. 1b) and T helper (CD4^+^ Th, Fig. 1c) cells after one month of treatment. Chemotherapy as well as radiotherapy is often accompanied by lymphopenia^27^. In the patient treated with docetaxel, there was little or no effect on the levels of T lymphocytes (Fig. 1, right panels black diamond). However, two other patients in the chemotherapy-group (black symbols in figures) displayed a transient reduction in all T lymphocyte populations investigated (Fig. 1, right panels). Of note, these patients received not only capecitabine (Xeloda; a fluoropyrimidine that is converted to 5-FU once ingested), but were also treated with radiation after the baseline samples were taken. Interestingly, the patient that received 5-FU in combination with epirubicin and cyclophosphamide (FEC) displayed a continuous increase in all T lymphocyte populations in circulation (Fig. 1, right panels, black circle). The effects are likely due to a general influence on all T lymphocyte sub-populations as the percentages of CD8^+^ T_c_/CTLs as well as CD4^+^ Th cells remain fairly constant, or even decrease, within the total lymphocyte population (all CD3^+^ cells) in the majority of patients including the patient that received FEC (Supplementary Fig. 2a,b). Similarly, the majority of patients displayed unaffected or modestly increased levels of regulatory T cells (Tregs) during treatment (Supplementary Fig. 2c). The patient with TNBC did, however, display a more pronounced increase (Supplementary Fig. 2c, right panels black box).

In order to assess more long-term effects, we followed five patients up to six months after treatment onset (Supplementary Fig. 3). CTLs and Th cells continued to decline within the total lymphocyte population in one patient treated with FEC, whereas a continuous increase in the same populations were observed for two patients treated with endocrine therapy (Supplementary Fig. 3b,c). The levels of regulatory T cells remained above baseline and even increased further for four of five patients (Supplementary Fig. 3d). Furthermore, as two patients received radiation therapy, we assessed the levels of circulating immune cells before and after radiation, but before initiating systemic therapy. Only very modest variations were observed for all lymphocyte populations analyzed (Supplementary Fig. 4a–e).

Altogether this indicates that T lymphocytes are only modestly affected during systemic treatment and could potentially be harnessed by future immunotherapeutic regimens.

### NK- and NKT cells are low at baseline but tend to increase during endocrine therapy

**Figure 2:**
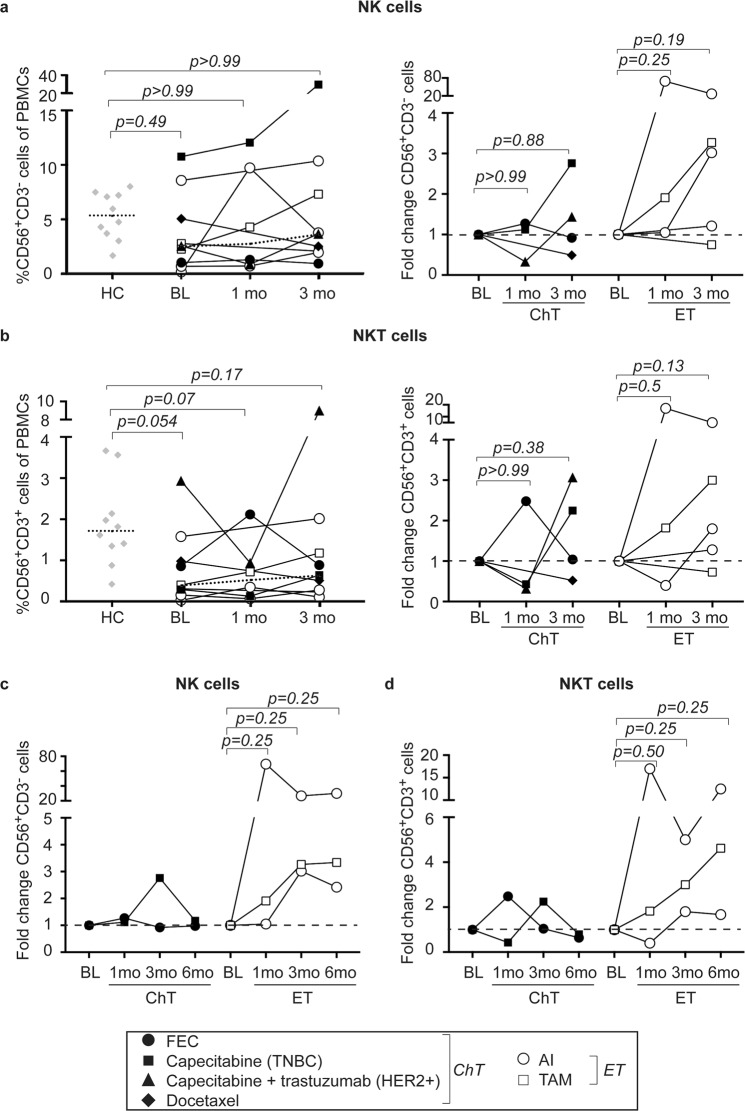
NK and NKT cells are low at BL but tend to increase with treatment. Flow cytometric analyzes of PBMCs from healthy controls (HC) and breast cancer patients before (baseline; BL), at 1 month, 3 months or at 6 months of treatment. (a,b, *left panels*) Percentages of CD56^+^CD3^−^ NK cells (**a**) or CD56^+^CD3^+^ NKT cells (**b**) of PBMCs. Dashed lines represent median. HC *n* = 10, BL *n* = 9, 1mo *n* = 6 and 3mo *n* = 9. Exact p-values, by Kruskal-Wallis with Dunn’s multiple comparison test, are indicated. (a,b, *right panels*) Fold change as compared to BL of NK (**a**) or NKT cells (**b**) upon treatment with chemotherapy (ChT, black symbols; BL *n* = 4, 1mo *n* = 3, 3 mo *n* = 4) or endocrine therapy (ET, white symbols; BL *n* = 5, 1mo *n* = 3, 3mo *n* = 5). Dashed line represents BL levels (set to 1). Exact p-values, by Kruskal-Wallis with Dunn’s multiple comparison test, are indicated. (**c,d**) Fold change as compared to BL of NK cells (**c**) or NKT cells (**d**) upon treatment with chemotherapy (ChT, black symbols, *n* = 2) or endocrine therapy (ET, white symbols, *n* = 3). Dashed line represents BL levels (set to 1). Exact p-values, by Wilcoxon signed-rank test, are indicated.

We next focused on circulating CD56^+^CD3^−^ NK and CD56^+^CD3^+^ NKT cells. Both populations are decreased in MBC patients at baseline as compared to healthy controls (Fig. 2a,b, left panels), although not statistically significant. The levels of NK- and NKT cells markedly increase over the course of the treatment in the majority of patients (Fig. 2a,b, right panels). One patient treated with chemotherapy (docetaxel) and one patient treated with endocrine therapy (tamoxifen) displayed lower levels at three months as compared to baseline (Fig. 2a,b, right panels). Interestingly, the levels of NK- as well as NKT cells remained high in comparison to baseline up to six months in patients treated with endocrine therapy, whereas they tended to normalize to baseline-levels in patients treated with chemotherapy (Fig. 2c,d).

As NK- and NKT cells are capable of recognizing and killing transformed cells without preceding antigen presentation, the observation that endocrine therapy may enrich for these populations will be of interest to look into in the future.

### Mo-MDSCs are enriched in metastatic breast cancer patients and decrease during systemic therapy

**Figure 3:**
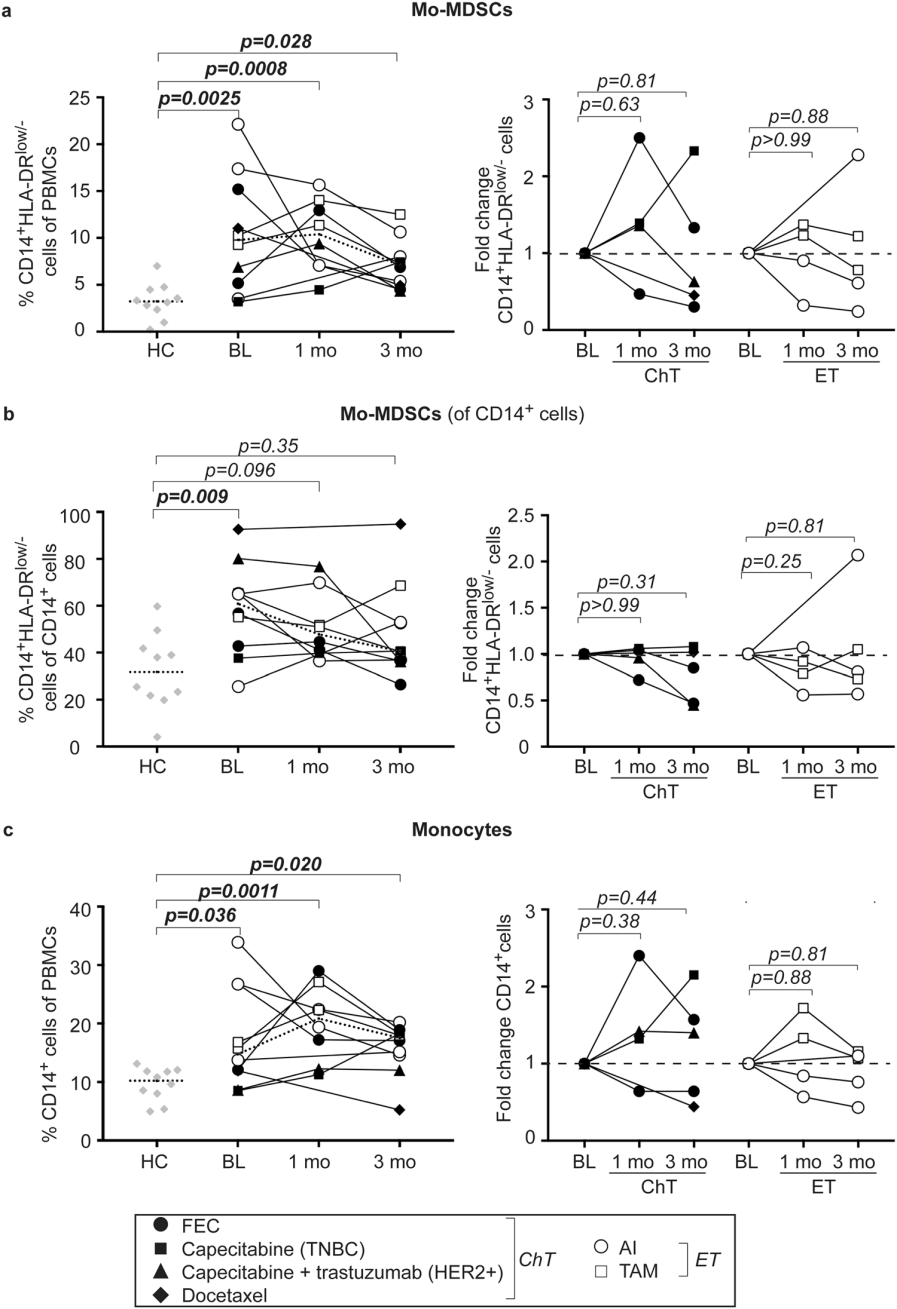
Mo-MDSCs are enriched at BL but decline during the course of treatment. Flow cytometric analyzes of PBMCs from healthy controls (HC) and breast cancer patients before (baseline; BL), at 1 month or at 3 months of treatment. (a–c, *left panels*) Percentages of CD14^+^HLA-DR^low/−^ Mo-MDSCs of PBMCs (**a**), Mo-MDSCs of CD14^+^ cells (**b**) or total CD14^+^ monocytes (**c**), depicted over time for individual patients. Dashed lines represent median. HC *n* = 10, BL *n* = 10, 1mo *n* = 8 and 3mo *n* = 10. Exact p-values, by Kruskal-Wallis with Dunn’s multiple comparison test are indicated, *p < 0.05, **p < 0.01, ***p < 0.001. (a–c, *right panels*) Fold change as compared to BL of Mo-MDSCs of PBMCs (**a**), Mo-MDSCs of CD14^+^ cells (**b**) or all CD14^+^ monocytes (**c**) upon treatment with chemotherapy (ChT, black symbols) or endocrine therapy (ET, white symbols). Dashed line represents BL levels (set to 1). BL *n* = 5, 1mo *n* = 4, 3mo *n* = 5. Exact p-values, by Wilcoxon signed-rank test, are indicated.

We were recently first to show that Mo-MDSCs (CD14^+^HLA-DR^low/−^Co-receptor^low/−^ cells) are enriched in the peripheral blood of breast cancer patients^23^. When analyzing the levels of Mo-MDSCs in MBC patients receiving systemic therapy, the levels were significantly higher also after one and three months of treatment, as compared to healthy controls (Fig. 3a). Compared to baseline, a transient increase could be observed at one month of treatment for the majority of patients (Fig. 3a, right panel). However, at three months the levels decreased dramatically for six out of ten patients. Similar trends were observed regardless treatment regimen and when looking at Mo-MDSCs within the total monocyte population (Fig. 3b) as well as all CD14^+^ monocytes (Fig. 3c). Interestingly, the patient with TNBC still displayed increasing levels of Mo-MDSCs at three months (Fig. 3a, right panel, black box). This may in part be related to the increased Mo-MDSC and monocyte levels observed after radiation therapy (Supplementary Fig. 4f–h).

**Figure 4:**
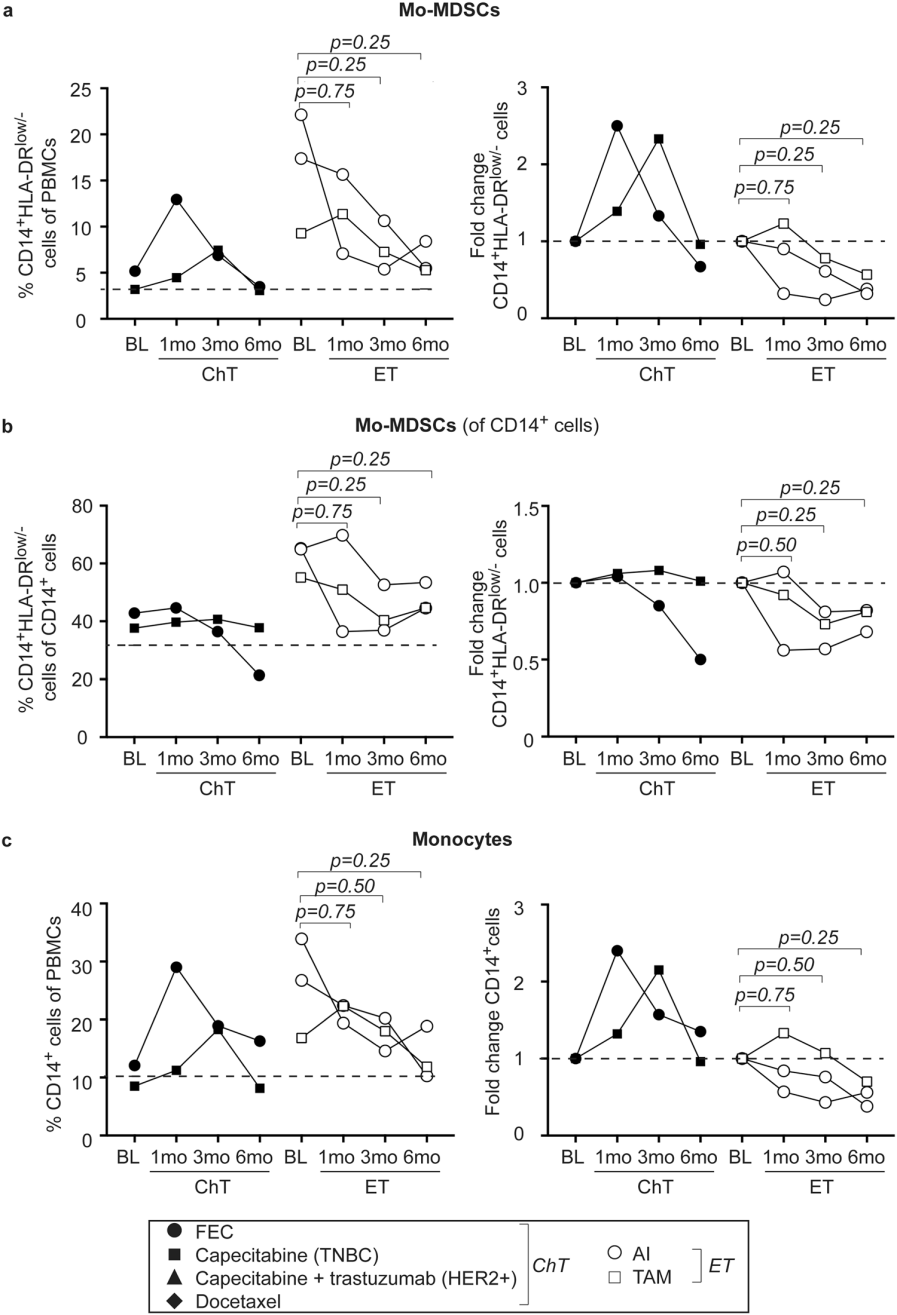
Mo-MDSC levels tend to normalize to healthy controls levels during systemic treatment. Flow cytometric analyzes of PBMCs from healthy controls (HC) and breast cancer patients before (baseline; BL), at 1 month, at 3 months or 6 months of treatment. (a–c, *left panels*) Percentages of CD14^+^HLA-DR^low/−^ Mo-MDSCs of PBMCs (**a**), Mo-MDSCs of CD14^+^ cells (**b**) or total CD14^+^ monocytes (**c**), depicted over time for individual patients treated with either chemotherapy (ChT, black symbols, *n* = 2) or endocrine therapy (ET, white symbols, *n* = 3). Dashed line represents median HC. Exact p-values, by Wilcoxon signed-rank test, are indicated. (a–c, *right panels*) Fold change as compared to BL of Mo-MDSCs of PBMCs (**a**), Mo-MDSCs of CD14^+^ cells (**b**) or all CD14^+^ monocytes (**c**) upon treatment with chemotherapy (ChT, black symbols *n* = 2) or endocrine therapy (ET, white symbols *n* = 3). Dashed line represents BL levels (set to 1). Exact p-values, by Wilcoxon signed-rank test are indicated.

When following five patients up to six months after treatment onset, Mo-MDSC as well as total monocyte levels tend to normalize to the level of healthy controls (Fig. 4, left panels). A substantial, albeit not significant, drop in the percentage of Mo-MDSCs was seen in patients treated with endocrine therapy (Fig. 4a, left panel). Furthermore, as compared to baseline levels, Mo-MDSCs in PBMCs as well as within the total CD14^+^ monocyte pool either continued or started to decline at six months (Fig. 4a,b, right panels). Similar trends are observed for monocytes (Fig. 4c, right panels).

Overall, Mo-MDSCs decrease in the peripheral blood during systemic therapy, though more consistent with endocrine therapy than with chemotherapy. This indicates that endocrine therapy may revert the enrichment of Mo-MDSCs in the peripheral blood of breast cancer patients. How this may be related to prognosis will be of interest to further look into in the future.

## Discussion

In the era of immunotherapy, a profound understanding of how specific immune cells are affected during conventional therapy is necessary. Although largely neglected, studies have indicated that some standard antitumoral agents also rely on their capacity to modulate the immune system for proper therapeutic efficacy^24^. In this pilot study, we evaluated a wide panel of circulating immune cells over time in patients with metastatic breast cancer receiving conventional antitumoral regimens. Although the patients monitored in this study reflect the general population well (with regards to hormone receptor status, age, as well as treatment) a larger study population is required to further validate the overall trends observed in this paper.

Chemotherapy is often accompanied by a transient lymphopenia. A transient reduction in T lymphocytes were observed in two out of four patients receiving chemotherapy. These patients were treated with capecitabine (converted to 5-FU once ingested) as well as radiotherapy. FEC and docetaxel, on the other hand, were rather associated with an increase in T lymphocytes or had no effect, respectively. Similar trends could also be observed for NK- and NKT cells with the main exception that the levels were restored and even increased at three months in the patients treated with capecitabine. These differences in chemotherapy effects on lymphocyte populations may theoretically also be influenced by the different dose regimens, *i*.*e*. capecitabine is an orally given 5-FU regimen based on a metronomic approach with a low dose ingested every day for two weeks and then one week without the drug. This approach differs from the more conventional high dose chemotherapy regimens (*i*.*e*, FEC) and can modulate the systemic immune response in different ways. A transient lymphopenia may be beneficial for the patient as this may activate homeostatic mechanisms that result in the proliferation of effector T cells such as tumor-reactive CD8^+^ CTLs^24,28^. Indeed, the levels of T lymphocytes were restored to baseline levels or above for the majority of the patients after three to six months of treatment.

Some agents such as cyclophosphamide and 5-FU have been suggested to selectively and transiently decrease the levels of immunosuppressive cells such as Tregs and MDSCs^24,28^. Indeed, Tregs were decreased or unaffected in three of four patients treated with chemotherapy. The dramatic increase seen in the fourth patient may be due to individual differences, to the TNBC phenotype of the tumor or to tumor progression. The same patient also displayed continuous increased levels of Mo-MDSCs, whereas the Mo-MDSC levels were markedly reduced for three of the other four patients. This is in line with previous findings showing that docetaxel treatment of primary human monocytes *in vitro* reduced the levels of Mo-MDSC-like cells while promoting the generation of pro-inflammatory M1 macrophages^26^. Circulating MDSCs, on the other hand, have previously been suggested to increase in breast cancer patients treated with doxorubicin and cyclophosphamide^22,29^. In both studies, granulocytic-MDSCs (G-MDSCs) were studied in patients with early stage breast cancer. In contrast to our results, Wesolowski *et al*. could not detect any variations in Mo-MDSCs^29^. This is likely due to differences in the patient groups being monitored; metastatic and early stage breast cancer, respectively, which is in line with our previous results^23^.

In our material, only two patients received cyclophosphamide (FEC). Four out of five treated with chemotherapy were, however, given 5-FU in some form (FEC or capecitabine). In mice bearing EL4 thymoma, 5-FU selectively deplete MDSCs thus restoring IFNγ production by CD8^+^ T cells^30^. Similar results were observed in 4T1-Neu-tumor bearing mice treated with docetaxel^31^. In patients, little is known about the impact of 5-FU on MDSCs. 5-FU in combination with folinic acid and oxaliplatin (FOLFOX) decreased the levels of G-MDSCs whereas 5-FU with folinic acid and CPT11 (FOLFIRI) tend to increase the MDSC levels in patients with colorectal cancer^32^. Thus, further clarification of the impact of 5-FU on MDSCs is required considering different dose regimens and combination treatments.

Information about how endocrine therapy affects circulating immune cells in cancer patients is scarce. In mice, tamoxifen was proposed to induce a shift from cellular Th1 to humoral Th2 immunity, while suppressing alloantigen- but not mitogen-induced T-cell proliferation *in vitro*^33–35^. Here, we observed a modest, but transient increase in T lymphocytes. This was especially pronounced for CD8^+^ CTLs after one month of treatment. Interestingly, patients treated with endocrine therapy also had an enrichment of NK and NKT cells in the peripheral blood. NK and NKT cells are well-known players in immunosurveillance and tumor rejection, and could potentially be exploited in future immunotherapies. Finally, a substantial reduction in the levels of Mo-MDSCs was observed in patients treated with endocrine therapy. This finding fits well with the observation that estrogens may drive MDSC accumulation^36^. To our knowledge, this is the first study to imply that MDSCs are affected by endocrine therapy. However, it is not possible to discriminate between direct effects of tamoxifen on MDSC accumulation and indirect effects mediated via tumor and host mechanisms respectively in this study. As the levels of circulating Mo-MDSCs correlate with disease progression, the underlying mechanisms and clinical implications to this observation will be of great interest to study in the future. The findings in this study are limited by the relatively small sample size but can be viewed as mainly hypothesis generating. Thus, larger studies regarding the impact of endocrine therapy on MDSCs, NK and NKT cells are warranted. We are currently analyzing different immune cell populations in a larger cohort and evaluating possible correlations to important clinicopathological factors and outcome.

To summarize, overall chemotherapy as well as endocrine therapy tend to have modest effects on lymphocyte populations, although endocrine therapy seems to induce an enrichment of NK- and NKT cells. In contrast, the Mo-MDSC levels decrease markedly in the majority of patients regardless of treatment type, although in particular in patients treated with endocrine therapy. Altogether these results indicate that the systemic immunosuppressive profile in patients with metastatic breast cancer tends to regress over the course of treatment, be it chemotherapy or endocrine therapy. Thus, combination treatment with standard antitumoral agents and immunotherapy will be interesting to follow.

## Materials and Methods

### Patient cohort and blood sampling

Peripheral blood immune cell populations were analyzed in ten patients, newly diagnosed with MBC, included in a prospective observational trial (ClinicalTrials.gov NCT01322893), during the course of systemic therapy. For details on this prospective observational trial, see previous publications^37^. Ethical permission has been obtained from the Research Ethics Committee at Lund University (Dnr 2012/689 for healthy blood donors, Dnr 2010/135 and Dnr 2011/748 for patients with breast cancer) and the study has been performed in accordance with the ethical standards in the Declaration of Helsinki. All participating patients and donors gave written informed consent.

Blood samples were taken at baseline (BL; before start of systemic therapy) and at one, three and six months during systemic therapy. Data from BL samples have previously been published as part of a larger dataset in refs^23,37^. See Table 1 for detailed patient information. Ten healthy blood donors were used as controls (90% female, mean age ± SEM; 39 ± 4 years). Peripheral blood from MBC patients and healthy controls was drawn in EDTA tubes and analyzed within 24 h as previously described^23^. Briefly, the peripheral blood was diluted 1:2 in PBS supplemented with 5 mM EDTA and 2.5% w/v sucrose and separated over Ficoll-Paque Plus (GE Healthcare, Uppsala, Sweden). The peripheral blood mononuclear cells (PBMCs) were collected.

### Flow cytometry

Freshly isolated PBMCs were washed once in FACS buffer (PBS supplemented with 3% fetal bovine serum and 0.1% sodium azide) and stained for flow cytometry. Antibodies used; CD14 clone M5E2, HLA-DR clone G46-6, CD86 clone IT2.2, CD163 clone GHI/61, CD3 clone HIT3a, CD4 clone RPA-T4, CD8 HIT8a, CD25 clone 2A3, CD127-biotin clone HIL-7R-M21, CD56 clone B159, all from BD Biosciences. All analyzes were performed on a FACS Calibur and gated on viable PBMCs using 7AAD dead exclusion stain (BD Biosciences, San Jose, CA, USA).

### Statistical analysis

Statistical analyzes on peripheral blood leukocyte populations were performed using the non-parametric Kruskal-Wallis with Dunn’s multiple comparison test for comparisons with healthy donors, or Wilcoxon matched-pairs signed rank test for comparisons with BL samples (GraphPad Prism 7). A p-value of < 0.05 was taken for significant.

### Précis

Conventional systemic therapy reduces the levels of circulating monocytic myeloid-derived suppressor cells in patients with metastatic breast cancer. NK and NKT cells tend to increase with endocrine therapy whereas lymphocytes are largely unaffected.

## Supplementary information

Supplementary Information

## References

[1] Global Burden of Disease Cancer, C (2017). Global, Regional, and National Cancer Incidence, Mortality, Years of Life Lost, Years Lived With Disability, and Disability-Adjusted Life-years for 32 Cancer Groups, 1990 to 2015: A Systematic Analysis for the Global Burden of Disease Study. JAMA Oncol.

[2] Torre LA (2015). Global cancer statistics, 2012. CA: a cancer journal for clinicians.

[3] Siegel RL, Miller KD, Jemal A (2017). Cancer Statistics, 2017. CA: a cancer journal for clinicians.

[4] Brewster AM (2008). Residual risk of breast cancer recurrence 5 years after adjuvant therapy. J Natl Cancer Inst.

[5] Early Breast Cancer Trialists’ Collaborative, G. (2015). Aromatase inhibitors versus tamoxifen in early breast cancer: patient-level meta-analysis of the randomised trials. Lancet.

[6] Beslija S (2009). Third consensus on medical treatment of metastatic breast cancer. Annals of oncology: official journal of the European Society for Medical Oncology/ESMO.

[7] Cardoso F (2018). 4th ESO-ESMO International Consensus Guidelines for Advanced Breast Cancer (ABC 4)dagger. Annals of oncology: official journal of the European Society for Medical Oncology/ESMO.

[8] Finn RS (2016). Palbociclib and Letrozole in Advanced Breast Cancer. The New England journal of medicine.

[9] Hortobagyi GN (2016). Ribociclib as First-Line Therapy for HR-Positive, Advanced Breast Cancer. The New England journal of medicine.

[10] Schmid P (2018). Atezolizumab and Nab-Paclitaxel in Advanced Triple-Negative Breast Cancer. The New England journal of medicine.

[11] Hanahan D, Coussens LM (2012). Accessories to the crime: functions of cells recruited to the tumor microenvironment. Cancer cell.

[12] DeNardo DG, Andreu P, Coussens LM (2010). Interactions between lymphocytes and myeloid cells regulate pro- versus anti-tumor immunity. Cancer metastasis reviews.

[13] Millrud CR, Bergenfelz C, Leandersson K (2017). On the origin of myeloid-derived suppressor cells. Oncotarget.

[14] Granger JM, Kontoyiannis DP (2009). Etiology and outcome of extreme leukocytosis in 758 nonhematologic cancer patients: a retrospective, single-institution study. Cancer.

[15] Takahashi R (2015). Prognostic significance of systemic neutrophil and leukocyte alterations in surgically treated endometrial cancer patients: a monoinstitutional study. Gynecol Oncol.

[16] Tavares-Murta BM (2010). Systemic leukocyte alterations are associated with invasive uterine cervical cancer. Int J Gynecol Cancer.

[17] Goedert JJ (2012). Peripheral blood immunologic phenotype of population-based breast cancer cases and matched controls. European journal of clinical investigation.

[18] Caras I (2004). Evidence for immune defects in breast and lung cancer patients. Cancer Immunol Immunother.

[19] Della Bella S (2003). Altered maturation of peripheral blood dendritic cells in patients with breast cancer. British journal of cancer.

[20] Melichar B, Touskova M, Dvorak J, Jandik P, Kopecky O (2001). The peripheral blood leukocyte phenotype in patients with breast cancer: effect of doxorubicin/paclitaxel combination chemotherapy. Immunopharmacol Immunotoxicol.

[21] Liyanage UK (2002). Prevalence of regulatory T cells is increased in peripheral blood and tumor microenvironment of patients with pancreas or breast adenocarcinoma. J Immunol.

[22] Diaz-Montero CM (2009). Increased circulating myeloid-derived suppressor cells correlate with clinical cancer stage, metastatic tumor burden, and doxorubicin-cyclophosphamide chemotherapy. Cancer Immunol Immunother.

[23] Bergenfelz C (2015). Systemic Monocytic-MDSCs Are Generated from Monocytes and Correlate with Disease Progression in Breast Cancer Patients. PLoS One.

[24] Zitvogel L (2008). The anticancer immune response: indispensable for therapeutic success?. J Clin Invest.

[25] Andre F (2013). Molecular pathways: involvement of immune pathways in the therapeutic response and outcome in breast cancer. Clin Cancer Res.

[26] Millrud CR, Mehmeti M, Leandersson K (2018). Docetaxel promotes the generation of anti-tumorigenic human macrophages. Experimental cell research.

[27] van Meir H (2017). Impact of (chemo)radiotherapy on immune cell composition and function in cervical cancer patients. Oncoimmunology.

[28] McDonnell AM, Nowak AK, Lake RA (2011). Contribution of the immune system to the chemotherapeutic response. Semin Immunopathol.

[29] Wesolowski R (2017). Circulating myeloid-derived suppressor cells increase in patients undergoing neo-adjuvant chemotherapy for breast cancer. Cancer Immunol Immunother.

[30] Vincent J (2010). 5-Fluorouracil selectively kills tumor-associated myeloid-derived suppressor cells resulting in enhanced T cell-dependent antitumor immunity. Cancer Res.

[31] Kodumudi KN (2010). A novel chemoimmunomodulating property of docetaxel: suppression of myeloid-derived suppressor cells in tumor bearers. Clin Cancer Res.

[32] Kanterman J (2014). Adverse immunoregulatory effects of 5FU and CPT11 chemotherapy on myeloid-derived suppressor cells and colorectal cancer outcomes. Cancer Res.

[33] Behjati S, Frank MH (2009). The effects of tamoxifen on immunity. Curr Med Chem.

[34] Bebo BF (2009). Treatment with selective estrogen receptor modulators regulates myelin specific T-cells and suppresses experimental autoimmune encephalomyelitis. Glia.

[35] Frank MH (2001). Specific MDR1 P-glycoprotein blockade inhibits human alloimmune T cell activation *in vitro*. J Immunol.

[36] Svoronos N (2017). Tumor Cell-Independent Estrogen Signaling Drives Disease Progression through Mobilization of Myeloid-Derived Suppressor Cells. Cancer discovery.

[37] Larsson AM (2018). Longitudinal enumeration and cluster evaluation of circulating tumor cells improve prognostication for patients with newly diagnosed metastatic breast cancer in a prospective observational trial. Breast cancer research: BCR.

