# Supplementary material for: Impact of systemic therapy on circulating leukocyte populations in patients with metastatic breast cancer
Source: Sci Rep. 2019 Sep 17;9:13451. doi: 10.1038/s41598-019-49943-y (PMC6748932; doi:10.1038/s41598-019-49943-y)
Supplement: Supplementary file 1 — Supplementary Information [file 41598_2019_49943_MOESM1_ESM.pdf]

# Impact of systemic therapy on circulating leukocyte populations in patients with metastatic breast cancer

**Anna-Maria Larsson[1,2], Anna Roxå[1], Karin Leandersson[3] and Caroline Bergenfelz[3,4*]**

Affiliations:

[1.] Department of Clinical Sciences Lund, Division of Oncology and Pathology, Lund University, Lund, Sweden

[2.] Department of Hematology, Oncology and Radiation Physics, Skåne University Hospital, Lund, Sweden

[3.] Department of Translational Medicine, Cancer Immunology, Lund University, Malmö 21428, Sweden

[4.] Department of Translational Medicine, Experimental Infection Medicine, Lund University, Malmö 21428, Sweden

[*] To whom correspondence should be addressed: Caroline Bergenfelz, +46-40-391027

E-mail: caroline.bergenfelz@med.lu.se

Supplementary Figure 1.

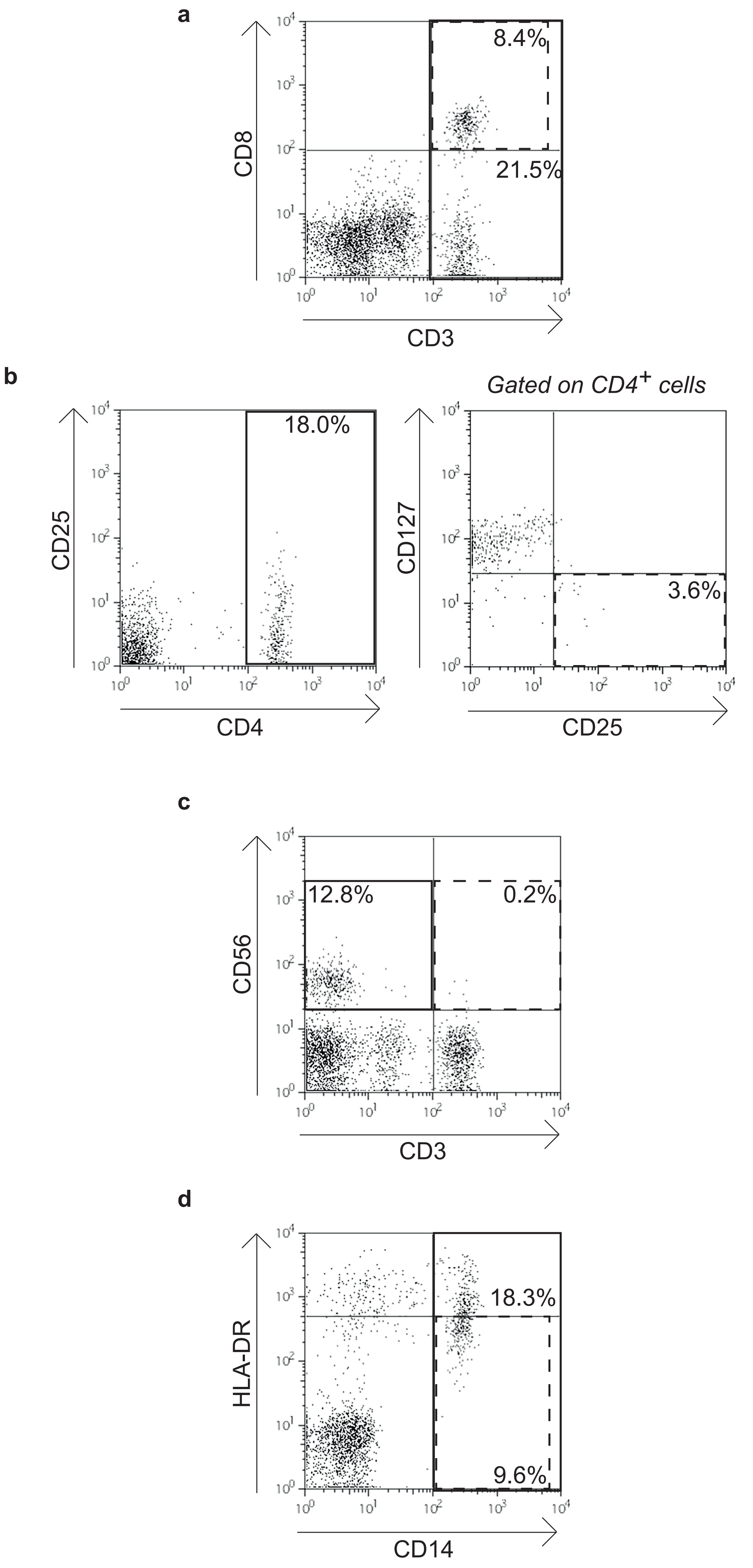

Supplementary Figure 2.

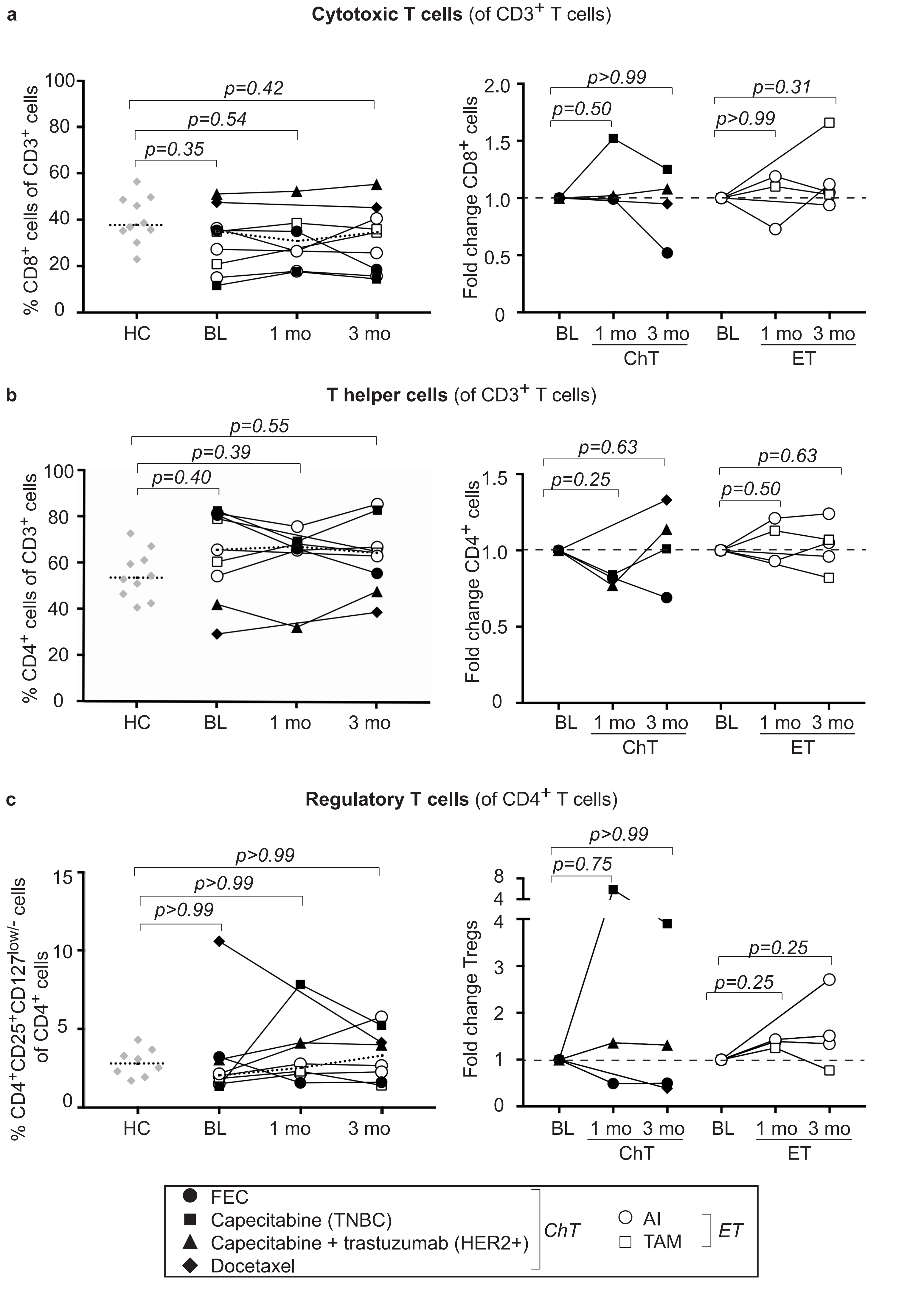

Supplementary Figure 3.

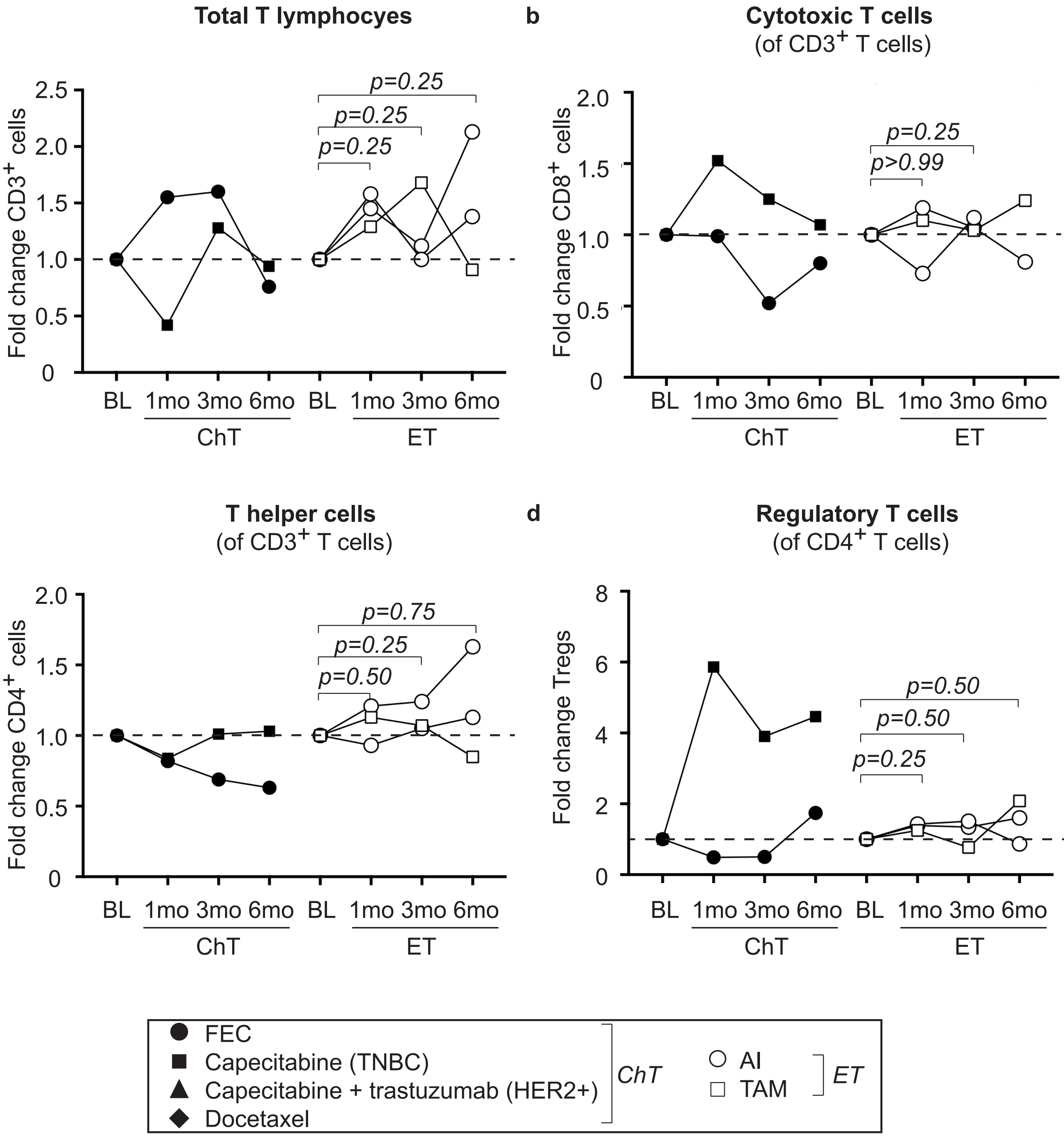

Supplementary Figure 4.

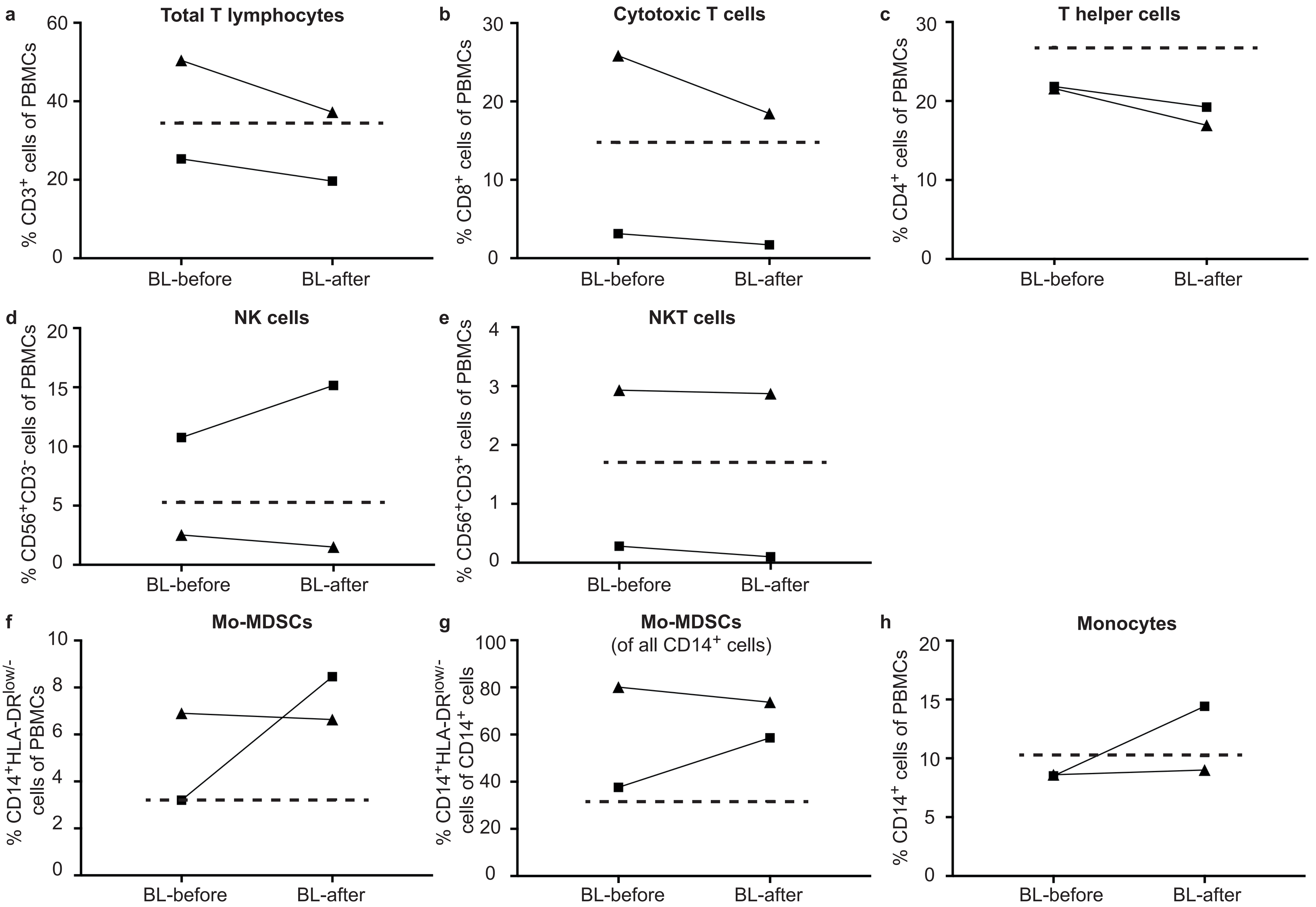

## Supplementary Figure Legends

**Supplementary Figure 1.** Representative dot plots and gating strategies of analyzed immune cell populations. All analyses are performed gated on viable (7AAD-negative) PBMCs. Percentage in gate shown. **a**. Total $CD3^+$ T lymphocytes (black box) and $CD8^+$ $T_c$/CTL (dashed box), **b.** $CD4^+$ Th cells (black box) and $CD4^+CD25^+CD127^{low/-}$ Tregs (dashed box). **c**. $CD56^+CD3^-$ NK cells (black box) and $CD56^+CD3^+$ NKT cells (dashed box). **d**. $CD14^+$ monocytes (black box) and $CD14^+$HLA-$DR^{low/-}$ Mo-MDSCs (dashed box).

**Supplementary Figure 2.** Flow cytometric analyzes of peripheral blood mononuclear cells (PBMCs) from healthy controls (HC) and breast cancer patients before (baseline; BL), at 1 month or at 3 months of treatment. (**a-b**, ***left panels***) Percentages of $CD8^+$ $T_c$/CTLs (a) or $CD4^+$ Th cells (b) of all $CD3^+$ T lymphocytes, depicted over time for individual patients. Dashed lines represent median HC $n$=10, BL $n$=9, 1mo $n$=6 and 3mo $n$=9. Exact p-values, by Kruskal-Wallis with Dunn's multiple comparison test, are indicated. (**a-b, *right panels***) Fold change as compared to BL of $CD8^+$ $T_c$/CTLs (a) or $CD4^+$ Th cells (b) upon treatment with chemotherapy (ChT, black symbols; BL $n$=4, 1mo $n$=3, 3mo $n$=4) or endocrine therapy (ET, white symbols; BL $n$=5, 1mo $n$=3, 3mo $n$=5). Dashed line represents BL levels (set to 1). Exact p-values, by Wilcoxon signed-rank test, are indicated. (**c, *left panel***) Percentages of $CD4^+CD25^+CD127^{low/-}$ Tregs of $CD4^+$ cells depicted over time for individual patients. Dashed lines represent median. HC $n$=10, BL $n$=8 1mo $n$=6 and 3mo $n$=8. Exact p-values, by Kruskal-Wallis with Dunn's multiple comparison test, are indicated. (**c, *right panel***) Fold change of Tregs as compared to BL upon treatment with chemotherapy (ChT, black symbols) or endocrine therapy (ET, white symbols). Dashed line represents BL levels (set to 1). BL $n$=4, 1mo $n$=3, 3mo $n$=4. Exact p-values, by Wilcoxon signed-rank test, are indicated.

**Supplementary Figure 3.** Flow cytometric analyzes of peripheral blood mononuclear cells (PBMCs) from breast cancer patients before (baseline; BL), at 1 month, at 3 months or at 6 months of treatment with chemotherapy (ChT, black symbols, *n*=2) or endocrine therapy (ET, white symbols, *n*=3). Fold change of total $CD3^+$ T lymphocytes of PBMCs (**a**), $CD8^+$ $T_c$/CTLs of $CD3^+$ cells (**b**), $CD4^+$ Th of $CD3^+$ cells (**c**) or Tregs of $CD4^+$ cells (**d**) as compared to BL. Dashed line represent BL levels (set to 1). Exact p-values, by Wilcoxon signed-rank test are indicated.

**Supplementary Figure 4.** Flow cytometric analyzes of peripheral blood mononuclear cells (PBMCs) from breast cancer patients before and after radiation therapy, and before onset of chemotherapy. Percentages of total $CD3^+$ T lymphocytes (**a**), $CD8^+$ $T_c$/CTLs (**b**), $CD4^+$ Th (**c**), $CD56^+CD3^-$ NK cells (**d**), $CD56^+CD3^+$ NKT cells (**e**), $CD14^+$HLA-$DR^{low/-}$ Mo-MDSCs (**f**), Mo-MDSCs of $CD14^+$ cells (**g**) and $CD14^+$ monocytes (**h**). Dashed lines represent median HC *n*=10.
